# Optical, Thermal, and Electrical Characterization of Polyvinyl Pyrrolidone/Carboxymethyl Cellulose Blend Scattered by Tungsten-Trioxide Nanoparticles

**DOI:** 10.3390/polym15051223

**Published:** 2023-02-28

**Authors:** Khadijah H. Alharbi, Walaa Alharbi, M. A. El-Morsy, M. O. Farea, A. A. Menazea

**Affiliations:** 1 Department of Chemistry, Science and Arts College, King Abdulaziz University, Rabigh 21911, Saudi Arabia; wnhalharbe@kau.edu.sa; 2 College of Science and Humanities in Al-Kharj, Physics Department, Plasma Technology and Material Science Unit, Prince Sattam Bin Abdulaziz University, Al-Kharj 11942, Saudi Arabia; elmorsym@yahoo.com; 3 Physics Department, Faculty of Science, University of Damietta, New Damietta 34517, Egypt; 4 Department of Physics, Faculty of Science, Mansoura University, Mansoura 35516, Egypt; mhd.omar1984@gmail.com; 5 Spectroscopy Department, Physics Research Institute, National Research Centre, Dokki, Giza 12622, Egypt; 6 Laser Technology Unit, Center of Excellent for Advanced Science, National Research Center, Dokki, Giza 12622, Egypt

**Keywords:** PVP, CMC, tungsten trioxide, XRD, TGA, PLA

## Abstract

The polymeric material polyvinyl pyrrolidine/carboxymethyl cellulose (PVP/CMC) was mixed with different quantities of tungsten-trioxide nanoparticles (WO_3_ NPs). The samples were created using the casting method and Pulsed Laser Ablation (PLA). The manufactured samples were analyzed by utilizing various methods. The halo peak of the PVP/CMC was located at 19.65°, confirming its semi-crystalline nature, as shown in the XRD analysis. FT-IR spectra of pure PVP/CMC composite and PVP/CMC composite incorporated with various contents of WO_3_ obtained a shift in band locations and change in intensity. Optical band gap was calculated via UV–Vis spectra, which decreased when increasing the laser-ablation time. Thermogravimetric analyses (TGA) curves showed that samples’ thermal stability had improved. The frequency-dependent composite films were used to determine AC conductivity of the generated films. When increasing the content of tungsten-trioxide nanoparticles, both (ε′) and (ε′′) increased. The incorporation of tungsten trioxide enhanced the ionic conductivity of PVP/CMC/WO_3_ nano-composite to a maximum of 10^−8^ S/c. It is expected that these studies will have a significant impact on several utilizations, such as energy storage, polymer organic semiconductors, and polymer solar cells.

## 1. Introduction

Mixing two or more polymers which mean blending polymers, it is possible to generate novel polymeric materials by new characterization with the necessary uses not found in one polymer alone, desirable characteristics, and a reasonable price [1]. Complexation between the segment chains of two polymers, such as charge transfer complexes, dipole-dipole, and forces’ hydrogen bonding, make them miscible [2]. Polyvinyl pyrrolidine (PVP) has a moderately conductive electrical conductivity and is suitable for a wide range of materials, since it has great stability. Polyvinyl pyrrolidine (PVP) has a rich charge transport and is considered a low-toxicity polymer [3].

Carboxymethyl cellulose (CMC) is a hydrophilic carboxyl group and a water-soluble hydrophobic polysaccharide backbone. CMC also has excellent film-forming properties because it is a low-cost, biodegradable, semi-crystalline, and ecologically friendly polymer [4]. Carboxymethyl cellulose (CMC) has been the subject of numerous studies aimed at advancing its application in electrolyte systems. Additionally, CMC has limited stability and miscibility, which is reflected in its performance when utilized in electrochemical devices [5]. An amorphous polymer must be added to CMC to increase its conductivity. PVP is used as a CMC partner in the manufacturing of composite materials and is expected to allow ionic mobility while minimizing scattering losses, making it suitable for various applications [6]. Another advantage of carbonyl groups in PVP is that they facilitate the interaction of cations with a broad range of inorganic nanoparticles, making it easier to introduce free mobile ions [7]. It is possible to change and enhance the physical, optical, electrical, and dielectric characterization of polymer composites by adding appropriate dopants [8].

Laser ablation (LA) has been considered one route to synthesis of different shapes of nanoparticles in different media (solutions and air) [9]. The mechanism of production of NPs by LA in solution could be concluded as being the following: there is a laser-beam incidence on the bulk material, and this material interacts with this laser beam and leads to an absorption of the laser by the material. After that, a plume of plasma is formed, then an expansion, then a production of shock waves, followed by a bubble production that could lead to the fabrication of nanoparticles in the liquid solutions [10]. Important factors that could affect on the produced nanoparticles include wavelength, pulse duration, energy of incident laser beam, and the solution medium which the nanoparticles fabricated through [11].

One of the most effective methods for enhancing the effectiveness of material properties, including structural, optical, mechanical, dielectric, and thermal characteristics, is the inclusion of metallic nanoparticles into polymer blends. Metals, metal oxides, and other nanostructure additions can be used to generate nanocomposites [12]. Over the last few years, transition metal oxides (TMOs) were explored for applications including electrical, optical, and mechanical characteristics. For the aforementioned applications, TMOs such as tungsten trioxide (WO_3_) are promising materials [13,14]. The insertion of these TMOs into polymers improves the electrical, thermal, and optical properties of the polymers. This improvement is due to the high interfacial interaction that these TMOs have between inorganic nanoparticles and organic components. Nowadays, enhancing the electrical conductivity of materials attracts enormous attention in terms of research due to this being a continuous, recyclable, and environmentally benign power source of solar energy [15]. The primary objective of this research is to manufacture varied concentrations of tungsten trioxide (WO_3_)-loaded PVP/CMC nanocomposite samples using a laser-ablation approach and to modify the electrical properties of PVP/CMC composite. LA is the easiest process for producing a nanoparticles-loaded-polymer-composite film. We discuss how to make various concentrations of tungsten trioxide (WO_3_)-loaded PVP/CMC composite samples and how to modify the electrical properties of PVP/CMC matrix.

## 2. Experimental Section

### 2.1. Chemicals

The blend includes polyvinyl pyrrolidone, which has been brought from ACROS, NJ, USA, in the form of crystals with a M.W. = 300,000 g mol^−1^. The carboxymethyl cellulose is used as a partner material with polyvinyl pyrrolidone to form a polymer blend. The molecular weight of the CMC provided by Sigma Aldrich, Germany was 172,000 g mol^−1^. The tungsten trioxide (WO_3_) was received from BDH chemical Ltd. pool in England (99.998%).

### 2.2. Preparation of (PVP/CMC/WO_3_)

The weight percent 70/30 mixtures of PVP/CMC were dissolved in UltraPure water, and the PVP and CMC mixture was stirred over for 4 days at 55 °C. The WO_3_ plate was put in a Piker containing 25 mL of the produced PVP/CMC composite solution. The used laser was a nanosecond Nd: YAG laser with a wavelength of 1064 nm. To synthesize PVP/CMC/WO_3_ nanocomposites, laser beam has been focused on the surface of tungsten plate with a convex length of 50 mm. The resulting PVP/CMC/WO_3_ nanocomposites were then casted in Petri dish for 72 h to get the desirable nanocomposites, which were subsequently examined by various methods. After drying the prepared solutions of each nanocomposite in glass Petri dishes, the films were ready for measurement.

### 2.3. Characterization

XRD scans with Cu K_α_ radiation were obtained using PANalytical PRO XRD, where λ = 0.1540 nm at 30 kV, and the 2θ was 5–80. Nicolet iS10 was used to obtain FT-IR spectra in the range of 4000 cm^−1^ to 400 cm^−1^. UV/Visible spectrum was detected using a spectrophotometer (Perkin–Elmer) with a wavelength range of 190–1000 nm. The films were thermally investigated with a Shimadzu TGA-50H. The electrical conductivity of the PVP/CMC mixture and the prepared PVP/CMC/WO_3_ nano-composite films were investigated using a HIOKI 3532 LCR meter automated and programmable in frequency range of 10 Hz–7 MHz.

## 3. Results and Discussion

### 3.1. X-ray Diffraction

**Figure 1:**
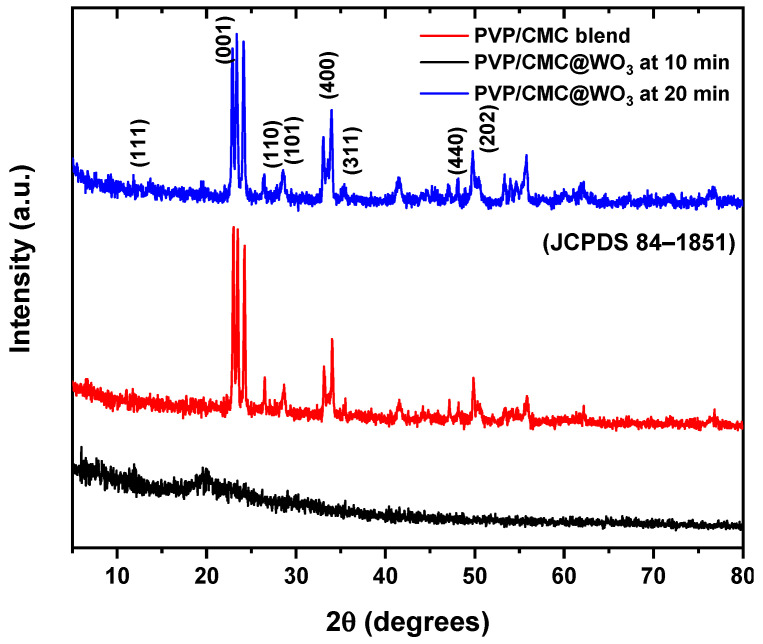
XRD pattern of polymer blend PVP/CMC and blend filled with WO_3_ at 10 min and 20 min laser-ablation time.

XRD analysis is most popular analytical route for illustrating the creation of WO_3_ nanoparticles and determining the crystal particle size. The XRD pattern of PVP/CMC and PVP/CMC/WO_3_ nanocomposite films at different ratios of tungsten dioxide nanoparticles are shown in Figure 1. The PVP/CMC blend’s XRD diffractogram is semicrystalline, with the major halo peak located at 19.65°, according to the Scherrer formula from the (111) plane. There is a tiny halo peak at 2θ = 13.75° and major halo peak at 2θ = 19.65° in the PVP/CMC/WO_3_ diffractogram. This is the nanocomposite sample’s PVP characteristic peak. Additional peaks occur when WO_3_ is incorporated into the PVP/CMC composite, and the intensity of the XRD peaks of the PVP/CMC/WO_3_ nanocomposite varies. The new diffraction peaks located at 2θ = 23.2°, 25.1°, and 34.1° are comparable to the monoclinic WO_3_, as reported by Liu 2015 [16]. The peaks can be indexed as (111), (001) and (110), which are characteristic peaks of crystal planes for WO_3_ [17]. The WO_3_ NPs were formed inside the PVP/CMC polymer blend by the complexation between WO_3_ and (C=O) group and the functional groups in PVP and (COO) group in CMC [18]. The reduction in intensity of peak which located at 2θ = 19.65° in nanocomposite samples also confirms these interactions, implying that the amorphous region of the nanocomposite samples has been raised. The inclusion of WO_3_ appears to change the average intersegmental spacing of the polymeric matrix just slightly, according to this reduction. Furthermore, it shows that the PVP/CMC blend and WO_3_ complexation occur in the amorphous area.

### 3.2. FTIR Technique

**Figure 2:**
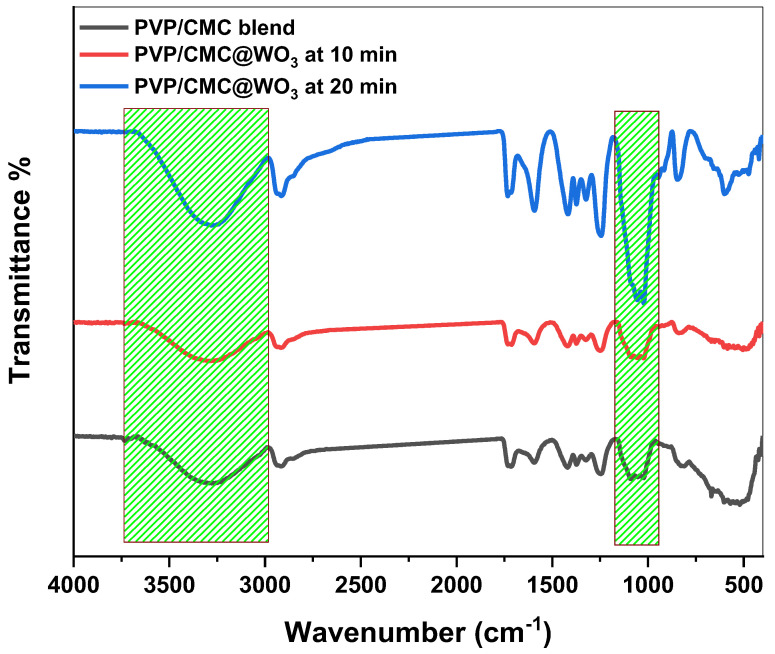
FT-IR band of polymer blend PVP/CMC and blend filled with WO_3_ at 10 min and 20 min laser-ablation time.

The physico-chemical changes that occurred in the PVP/CMC/WO_3_ nano-composite films due to the interaction which occurs between pure blend and tungsten trioxide nanoparticles were determined using FT-IR. Figure 2 shows FTIR bands of the PVP/CMC blend before and after incorporation with WO3 at 10 min and 20 min. The following vibrational frequencies may be given to some of the characteristic bands in the PVP/CMC blend: 3291 cm^−1^ for OH (hydroxyl function), 2919 cm^−1^ for C–H stretching, 1705 cm^−1^ for C–O stretching, and 1591 cm^−1^ for the N–H stretching vibration. The CH_2_ scissoring vibrational motion was ascribed to band at 1421 cm^−1^ [19]. The band detected at 1663 cm^−1^ could be ascribed to carbonyl group (C=O) of PVP, whereas carboxylate (COO) and (CH_2_–O–CH_2_) groups of CMC were detected at 1596 cm^−1^ and 968 cm^−1^, respectively, in the PVP/CMC spectra [20]. This confirms that PVP and CMC are interact via the formation of hydrogen bonds. The band at 617 cm^−1^ and 942 cm^−1^ is related to the W-O-W stretching vibration of tungsten trioxide nanoparticles [18]. Figure 2 shows that when the laser ablation began, the band intensities increased, especially after 20 min of laser ablation, and the typical broadband at 3291 cm^−1^ was completely increased for all films. In the case of other bands, their intensities are either shifted or increased for doped films. Hydrogel samples were subjected to some interactions, and their chemical structures changed as a result of these significant modifications in bands.

### 3.3. Optical Parameters

**Figure 3:**
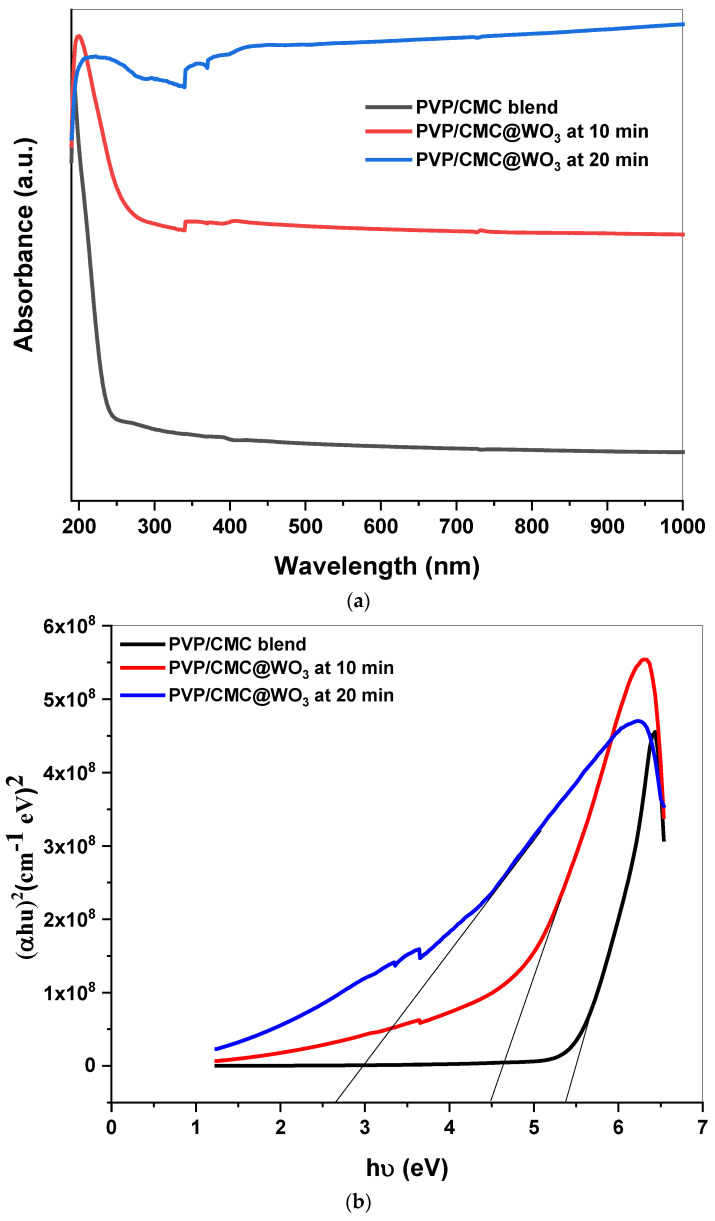

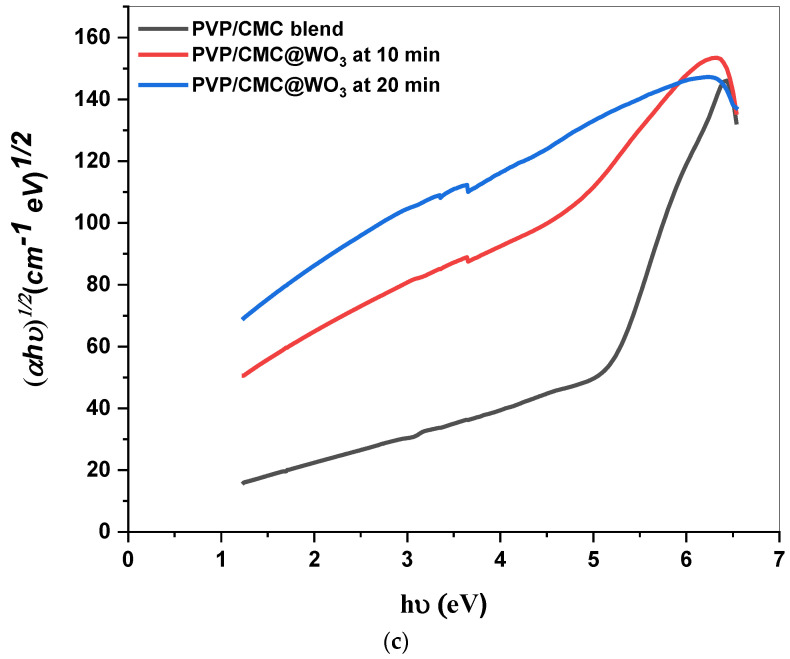
(**a**) Absorption spectrum of PVP/CMC and blend filled with WO_3_ at 10 min and 20 min. (**b**) Plot of αhυ^2^ versus hυ of PVP/CMC and blend scattered by WO_3_ at 10 min and 20 min. (**c**) Plot of αhυ^1/2^ versus hυ of PVP/CMC and blend scattered by WO_3_ at 10 min and 20 min.

The optical characteristics of PVP/CMC/WO_3_ ternary polymeric composites with different transition metal oxide donors have been obtained in Figure 3a–c. Figure 3a illustrates the absorption performance, which varies with the addition of tungsten trioxide. The transition mode (n → π*) could be indicated by the peak centered near 201 nm. The tungsten trioxide contribution caused a slight shift, which could hint at a significant spreading of nanoparticles on the copolymer, in addition to structural changes that generate evident variances inside the *E_g_*, as shown in Figure 3b,c. In addition, the disappearing of noticeable bands in visible reign illustrates that this composite was transparent. Furthermore, the contribution of tungsten trioxide through the blend is allocated to the low peak of about 340 nm [21]. Despite its low intensity, the tungsten-trioxide peak increases as the WO_3_ filling level increases. The energy gap may be calculated using the below equation [22,23]:

(1) $$\alpha h\upsilon =B{\left(h\upsilon -E_{g\;}\right)}^{r}$$

where $E_{g\;}\mathrm{is}\;\mathrm{the}\;\mathrm{energy}\;\mathrm{gap}$ , *h* υ represents the energy of the photon, and *B* represents the constant. The type of transition is indicated by the power *r*. Values of (direct and indirect) energy gap were listed in Table 1. A smaller bandgap is seen during blend transitions, and this band gap decreases as the amount of WO_3_ contributes to the overall band gap. Furthermore, the decrease in the energy gap implies better crystallographic ordering. This improvement may assist in the electrical localization between the PVP/CMC and the WO_3_.

### 3.4. Thermal Analysis

**Figure 4:**
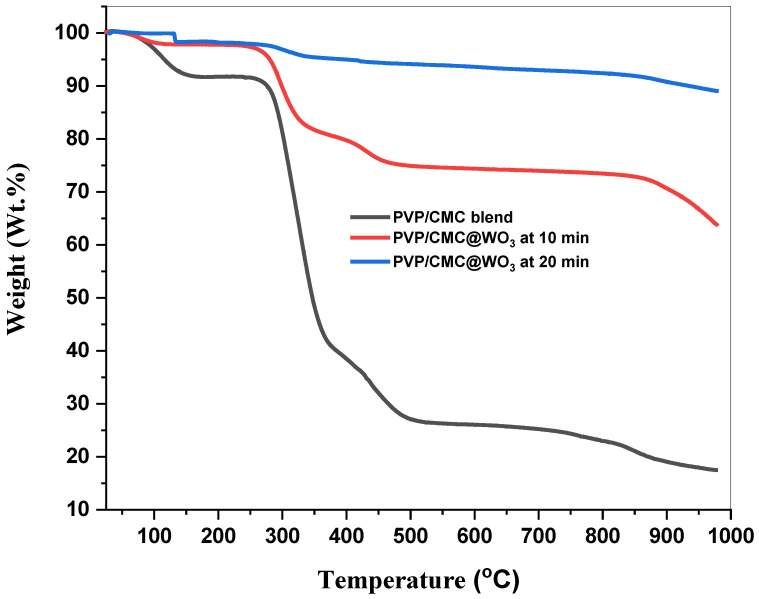
TGA curves of PVP/CMC and blend filled with WO_3_ at 10 min and 20 min.

The influence of tungsten trioxide on the thermal degradation behaviors of the PVP/CMC matrix was examined using thermogravimetric analyses. TGA graphs of the blend (PVP/CMC) mixed with WO_3_ at various laser-ablation period have been illustarted in Figure 4. The initial weight loss for PVP/CMC in the absence of tungsten-trioxide nanoparticles was observed at 100–285 °C, whereas the initial weight loss for PVP/CMC-WO_3_ nanocomposite films was observed at 278 due to the presence of WO_3_ in the films, which was produced by the breakdown of chains in PVP/CMC blend. As shown in Figure 4, the generated nano-composite are stable in all measurement range (up to 850 °C for the second sample), indicating that the fabricated films were stable thermally and was enhanced by raising the tungsten-trioxide concentration. The PVP/CMC blend’s split C-C backbone produced the second loss at 850 °C, with a total weight loss of about 70 wt.% at 980 °C. The incorporation of WO_3_ into the manufactured films enhanced their thermal stability, as shown by these studies. The thermal disintegration of PVP/CMC blends with varied concentrations shifts to a higher temperature than the PVP/CMC blend, suggesting that the indicated chemical reaction between tungsten trioxide and the PVP/CMC blend improves the thermal stability of the produced films [24]. In addition, it was revealed that increasing the amount of WO_3_ in the PVP/CMC mixture improved thermal properties of nano-composite, particularly in the last sample, which remained thermally stable up to 980 °C.

### 3.5. AC Conductivity

**Figure 5:**
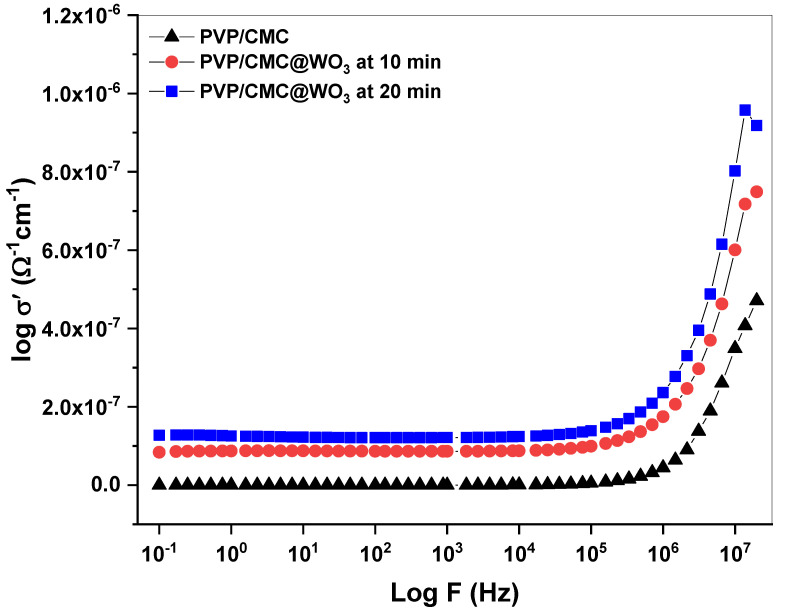
The difference of $\sigma_{\mathrm{ac}}$ with the frequency for the PVP/CMC-WO_3_ at two laser-ablation times (10 and 20 min).

Figure 5 depicts the relationship between log (σ) and log (f) for a composition of PVP/CMC and PVP/CMC/WO_3_ with various contents of tungsten-trioxide nanoparticles. Due to a larger energy barrier, the charge carriers’ numbers for a high relaxation time responding to the small frequencies zone might be reduced in a low conductivity at a low frequency. Due to their high conductivity, number of charge carriers with a low barrier was high and more sensitive to a high frequency. At lower frequencies, the hopping mechanism, as shown in Figure 5. There was a linear between log (σ) and log (f) at lower frequencies, but there was none at a high frequency, and there appeared to be a plateau. Using Equation (2), the conductivity of the samples, which is dependent on frequency dispersion ( ω ), can be calculated.

(2) $$\sigma_{\mathrm{ac}}=\sigma_{\mathrm{dc}}+A\omega^{S}$$

A is a constant, ω is usually proven to be dependent on the temperature, and $\sigma_{\mathrm{ac}}\;$ is the AC conductivity. The frequency exponent (S) is calculated using slopes of the straight lines produced by the relation between log $\sigma_{\mathrm{ac}}$ versus log (f) in the higher-frequency region. The frequency exponent values range from 0.81 to 20.52. These results correspond with previous studies, which are typical amorphous semiconductor characteristics [25,26].

### 3.6. Dielectric Studies

**Figure 6:**
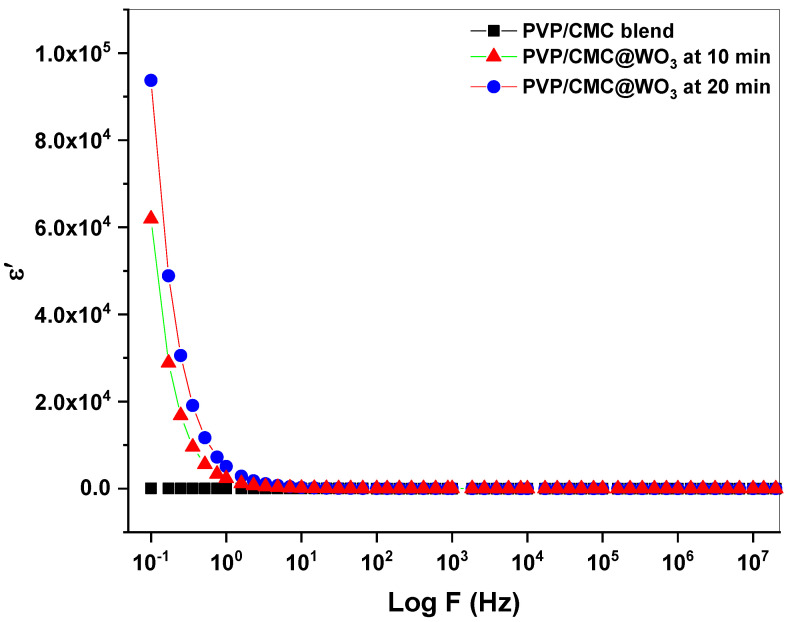
The real part (ε′) versus log *f* for PVP/CMC and PVP/CMC-WO_3_ at two laser-ablation times (10 and 20 min).

**Figure 7:**
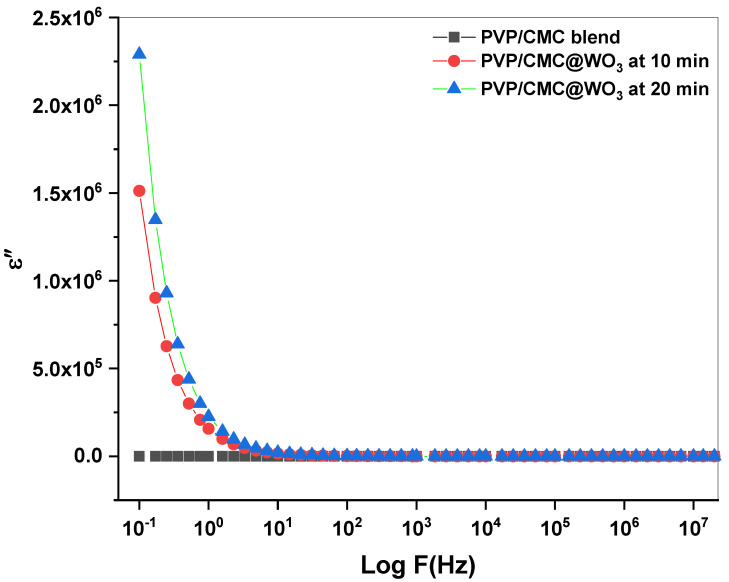
The imaginary part (ε″) versus log *f* for PVP/CMC and PVP/CMC-WO_3_ at two laser-ablation times (10 and 20 min).

The frequency (f) affects the real part (ε′) and imaginary part (ε″) of dielectric. This relationship demonstrates the importance of the WO_3_ transition metal in the changes to the physical properties of PVP/CMC. Figure 6 and Figure 7 show how the frequency fluctuates in εʹ and ε′′. As frequency of the prepared films rises, a reduction in ε′ and ε′′ is noticeable. With a rising frequency, the values of εʹ and ε′′ reduces substantially until get a constant value at a high frequency. Due to the polarization loss induced by charge accumulation, the ε′ and ε′′ values decrease [27,28]. The real-part and imaginary-part values increase with an increasing time of laser ablation for all frequency ranges. The higher ε′ and ε′′ at a high laser-ablation time might be associated by an raising in accumulated charge due to the polarization in the interaction between tungsten trioxide nanoparticles and PVP/CMC [29].

As the graphs illustrate, a rise in εʹ correlates with an increase in film conductivity. The dielectric loss value exhibits a dispersion at lower frequencies, but it continues to drop at higher frequencies and reaches zero for all samples. This is because charges may affect the direction of the field at a lower frequency related to a large amount of available time, but this contribution reduces as the frequencies raising, leading to a decrease in dielectric loss. At lower frequencies, the rise in charge mobility produced by the raise room temperature [18].

### 3.7. Argand Plot Measurements

**Figure 8:**
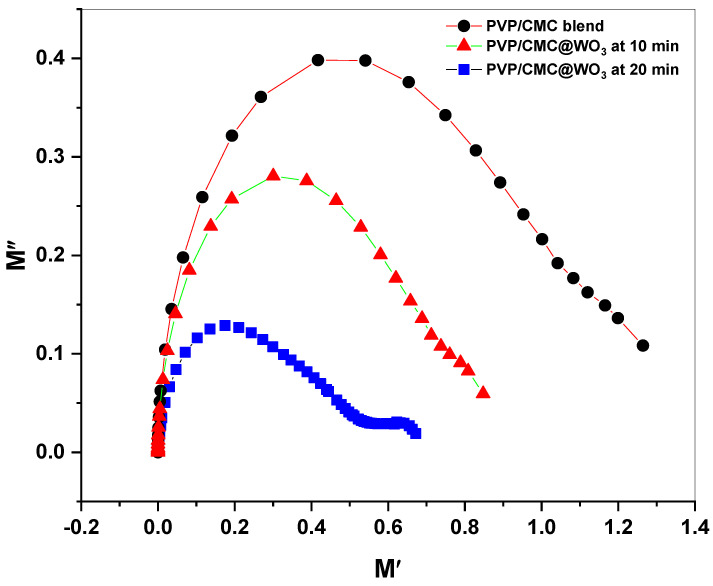
Argand plot of PVP/CMC and PVP/CMC -WO_3_ at two laser-ablation times (10 and 20 min).

The relaxation process in polymeric materials was due to ion conductivity, as shown in Argand illustration. The Argand plot for PVP/CMC/WO_3_ nanocomposite films at 0.0 min, 10 min and 20 min is shown in Figure 8. The relaxation time distribution of the polymers deviates from the semi-circular structure. The small semicircular arcs of the nanocomposite polymers are all present. The center of arc that was found in M′ axis, represents the electric relaxation (X-axis). Length of depressed semi-circular arc has been related to the electrical properties of films that were produced [30]. Figure 8 indicates that a decrease in the length of the arc is due to a better concertation of WO_3_ NPs in the polymer nanocomposite, increasing conductivity. Due to the addition of nanofiller, polymer nanocomposites with high content of tungsten trioxide nanoparticles exhibit a greater conductivity than other compositions. The Argand plot attempts to shift closer to center as the laser-ablation time in PVP/CMC rises. Furthermore, PVP/CMC/WO_3_ films are naturally non-Debye. The non-Debye character is due to a variety of polarization processes and relaxation processes as well as numerous interactions between dipoles and ions.

## 4. Conclusions

Laser ablation (LA) has been utilized to fabricate tungsten-trioxide NPs. The casting process was successful in producing a PVP/CMC composite doped with WO_3_. XRD measurements depict that the amorphousness of the samples varies with the addition of tungsten trioxide. The complexation between PVP/CMC and WO_3_ NPs was verified by FTIR analysis. The values of the energy gap (E_g_) for all produced samples decreased as the tungsten-trioxide-nanoparticle concentration increased. In comparison to the PVP/CMC polymer blend, the thermal stability of PVP/CMC/WO_3_ increased, demonstrating the favorable structural modifications brought about by the incorporation of WO_3_. The incorporation of WO_3_ into the PVP/CMC matrix enhanced AC-conductivity values. These nanocomposite samples might be employed for energy-storage devices.

## Figures and Tables

**Table 1:** The band gap [E_g_^in^ and E_g_^d^] values for polymer blends PVP/CMC and blends doped with WO_3_ at 10 and 20 min.

Samples	E_g_^d^ (eV)	E_g_^in^ (eV)
PVP/CMC	5.43	4.91
PVP/CMC/WO_3_ at 10 min	4.52	3.65
PVP/CMC/WO_3_ at 10 min	2.61	1.48

## Data Availability

Not applicable.

## References

[1] Meikhail M., Meikhail M.S., Oraby A.H., Farea M.O., Abdelghany A.M. (2014). Spectroscopic studies of PVA/PEO hydrogel filled with cesium chloride. Res. J. Pharm. Biol. Chem. Sci..

[2] Ramesan M.T., Varghese M., Periyat P. (2018). Silver-doped zinc oxide as a nanofiller for development of poly (vinyl alcohol)/poly (vinyl pyrrolidone) blend nanocomposites. Adv. Polym. Technol..

[3] Kochi R., Crasta V., Kumar R., Shetty G. (2019). Study of structural, optical and photoluminescence properties of ZnO doped PVA/PVP nanocomposite. AIP Conf. Proc..

[4] Saadiah M., Zhang D., Nagao Y., Muzakir S., Samsudin A. (2019). Reducing crystallinity on thin film based CMC/PVA hybrid polymer for application as a host in polymer electrolytes. J. Non-Cryst. Solids.

[5] Mazuki N.F., Fuzlin A.F., Saadiah M.A., Samsudin A.S. (2019). An investigation on the abnormal trend of the conductivity properties of CMC/PVA-doped NH4Cl-based solid biopolymer electrolyte system. Ionics.

[6] Kumar K.K., Ravi M., Pavani Y., Bhavani S., Sharma A.K., Rao V.N. (2014). Investigations on PEO/PVP/NaBr complexed polymer blend electrolytes for electrochemical cell applications. J. Membr. Sci..

[7] Koduru H., Scarpelli F., Marinov Y.G., Hadjichristov G.B., Rafailov P.M., Miloushev I.K., Petrov A.G., Godbert N., Bruno L., Scaramuzza N. (2018). Characterization of PEO/PVP/GO nanocomposite solid polymer electrolyte membranes: Microstructural, thermo-mechanical, and conductivity properties. Ionics.

[8] Pravakar O., Siddaiah T., Ramacharyulu PV R.K., Gopal N.O., Ramu C., Nagabhushana H. (2019). Spectroscopic, thermal, structural and electrical studies on VO2+ ions doped PVA/MAA: EA polymer blend films. J. Sci. Adv. Mater. Devices.

[9] Tommalieh M.J., Awwad N.S., Ibrahium H.A., Menazea A.A. (2021). Characterization and electrical enhancement of PVP/PVA matrix doped by gold nanoparticles prepared by laser ablation. Radiat. Phys. Chem..

[10] Menazea A.A., Awwad N.S., Ibrahium H.A., Ahmed M.K. (2020). Casted polymeric blends of carboxymethyl cellulose/polyvinyl alcohol doped with gold nanoparticles via pulsed laser ablation technique; morphological features, optical and electrical investigation. Radiat. Phys. Chem..

[11] Ismail A.M., El-Newehy M.H., El-Naggar M.E., Moydeen A.M., Menazea A.A. (2020). Enhancement the electrical conductivity of the synthesized polyvinylidene fluoride/polyvinyl chloride composite doped with palladium nanoparticles via laser ablation. J. Mater. Res. Technol..

[12] Kumar N.B.R., Crasta V., Bhajantri R.F., Praveen B.M. (2014). Microstructural and mechanical studies of PVA doped with ZnO and WO3 composites films. J. Polym..

[13] Ashraf S., Blackman C.S., Palgrave R.G., Parkin I.P. (2007). Aerosol-assisted chemical vapour deposition of WO3 thin films using polyoxometallate precursors and their gas sensing properties. J. Mater. Chem..

[14] Manno D., Serra A., Di Giulio M., Micocci G., Tepore A. (1998). Physical and structural characterization of tungsten oxide thin films for NO gas detection. Thin Solid Film..

[15] Liang G., Chen X., Ren D., Jiang X., Tang R., Zheng Z., Su Z., Fan P., Zhang X., Zhang Y. (2021). Ion doping simultaneously increased the carrier density and modified the conduction type of Sb2Se3 thin films towards quasi-homojunction solar cell. J. Mater..

[16] Liu F., Chen X., Xia Q., Tian L., Chen X. (2015). Ultrathin tungsten oxide nanowires: Oleylamine assisted nonhydrolytic growth, oxygen vacancies and good photocatalytic properties. RSC Adv..

[17] Khan H.U., Tariq M., Shah M., Ullah S., Ahsan A.R., Rahim A., Ismail I. (2021). Designing and development of polyvinylpyrrolidone-tungsten trioxide (PVP-WO3) nanocomposite conducting film for highly sensitive, stable, and room temperature humidity sensing. Mater. Sci. Semicond. Process..

[18] Abdelghany A., Oraby A., Farea M. (2019). Influence of green synthesized gold nanoparticles on the structural, optical, electrical and dielectric properties of (PVP/SA) blend. Phys. B Condens. Matter.

[19] Roy N., Saha N., Kitano T., Saha P. (2012). Biodegradation of PVP–CMC hydrogel film: A useful food packaging material. Carbohydr. Polym..

[20] Morsi M.A., Rajeh A., Menazea A.A. (2019). Nanosecond laser-irradiation assisted the improvement of structural, optical and thermal properties of polyvinyl pyrrolidone/carboxymethyl cellulose blend filled with gold nanoparticles. J. Mater. Sci. Mater. Electron..

[21] Franco D., De Plano L., Rizzo M., Scibilia S., Lentini G., Fazio E., Neri F., Guglielmino S., Mezzasalma A. (2020). Bio-hybrid gold nanoparticles as SERS probe for rapid bacteria cell identification. Spectrochim. Acta Part A Mol. Biomol. Spectrosc..

[22] Farea M., Abdelghany A., Meikhail M., Oraby A. (2020). Effect of cesium bromide on the structural, optical, thermal and electrical properties of polyvinyl alcohol and polyethylene oxide. J. Mater. Res. Technol..

[23] Chen S., Liu T., Chen M., Ishaq M., Tang R., Zheng Z., Su Z., Li X., Qiao X., Fan P. (2022). Crystal growth promotion and interface optimization enable highly efficient Sb2Se3 photocathodes for solar hydrogen evolution. Nano Energy.

[24] Abdelrazek E., Elashmawi I., El-Khodary A., Yassin A. (2010). Structural, optical, thermal and electrical studies on PVA/PVP blends filled with lithium bromide. Curr. Appl. Phys..

[25] Barrau S., Demont P., Peigney A., Laurent C., Lacabanne C. (2003). DC and AC conductivity of carbon nanotubes− polyepoxy composites. Macromolecules.

[26] Krstic V., Muster J., Duesberg G., Philipp G., Burghard M., Roth S. (2000). Electrical transport in single-walled carbon nanotube bundles embedded in Langmuir–Blodgett monolayers. Synth. Met..

[27] Rajeh A., Ragab H., Abutalib M. (2020). Co doped ZnO reinforced PEMA/PMMA composite: Structural, thermal, dielectric and electrical properties for electrochemical applications. J. Mol. Struct..

[28] Farea M., Abdelghany A., Oraby A. (2020). Optical and dielectric characteristics of polyethylene oxide/sodium alginate-modified gold nanocomposites. RSC Adv..

[29] Sengwa R., Choudhary S. (2010). Investigation of correlation between dielectric parameters and nanostructures in aqueous solution grown poly (vinyl alcohol)-montmorillonite clay nanocomposites by dielectric relaxation spectroscopy. Express Polym. Lett..

[30] Jeyabanu K., Siva V., Nallamuthu N., Selvanayagam S., Bahadur S.A., Manikandan A. (2018). Investigation of electrochemical studies of magnesium ion conducting poly (vinyl alcohol)–poly (vinyl pyrrolidone) based blend polymers. J. Nanosci. Nanotechnol..

