# Correction: Alharbi et al. Optical, Thermal, and Electrical Characterization of Polyvinyl Pyrrolidone/Carboxymethyl Cellulose Blend Scattered by Tungsten-Trioxide Nanoparticles. *Polymers* 2023, *15*, 1223

**DOI:** 10.3390/polym17010012

**Published:** 2024-12-25

**Authors:** Khadijah H. Alharbi, Walaa Alharbi, M. A. El-Morsy, M. O. Farea, A. A. Menazea

**Affiliations:** 1 Department of Chemistry, Science and Arts College, King Abdulaziz University, Rabigh 21911, Saudi Arabia; 2 College of Science and Humanities in Al-Kharj, Physics Department, Plasma Technology and Material Science Unit, Prince Sattam Bin Abdulaziz University, Al-Kharj 11942, Saudi Arabia; 3 Physics Department, Faculty of Science, University of Damietta, New Damietta 34517, Egypt; 4 Department of Physics, Faculty of Science, Mansoura University, Mansoura 35516, Egypt; 5 Spectroscopy Department, Physics Research Institute, National Research Centre, Dokki, Giza 12622, Egypt; 6 Laser Technology Unit, Center of Excellent for Advanced Science, National Research Center, Dokki, Giza 12622, Egypt

## Error in Figure

In the original publication [1], there was a mistake in Figure 1 as published. The figure consists of three curves, but one of these curves was repeated by mistake. The corrected figure appears below. The authors apologize for any inconvenience caused and state that the scientific conclusions are unaffected. This correction was approved by the Academic Editor. The original publication has also been updated.




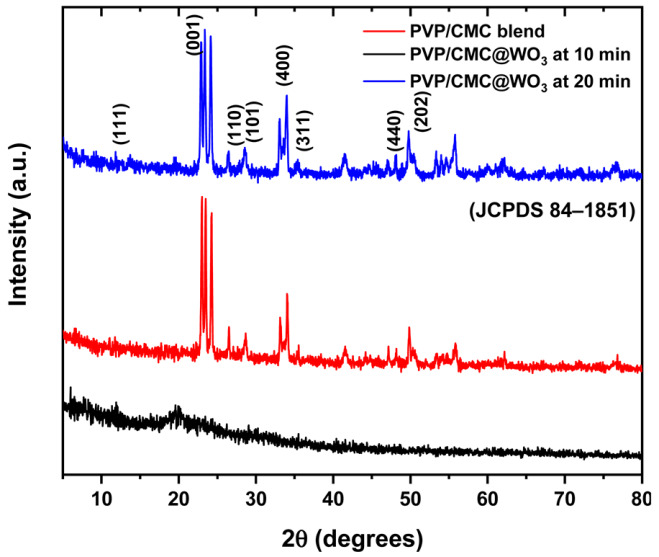


**Figure 1.** XRD pattern of polymer blend PVP/CMC and blend filled with WO_3_ at 10 min and 20 min laser-ablation time.


